# 18 kDa translocator protein positron emission tomography facilitates early and robust tumor detection in the immunocompetent SB28 glioblastoma mouse model

**DOI:** 10.3389/fmed.2022.992993

**Published:** 2022-10-17

**Authors:** Laura M. Bartos, Sabrina V. Kirchleitner, Jens Blobner, Karin Wind, Lea H. Kunze, Adrien Holzgreve, Lukas Gold, Artem Zatcepin, Zeynep Ilgin Kolabas, Selin Ulukaya, Lorraine Weidner, Stefanie Quach, Denise Messerer, Peter Bartenstein, Joerg C. Tonn, Markus J. Riemenschneider, Sibylle Ziegler, Louisa von Baumgarten, Nathalie L. Albert, Matthias Brendel

**Affiliations:** ^1^Department of Nuclear Medicine, University Hospital of Munich, LMU Munich, Munich, Germany; ^2^Department of Neurosurgery, University Hospital of Munich, LMU Munich, Munich, Germany; ^3^Helmholtz Center, Institute for Tissue Engineering and Regenerative Medicine (iTERM), Munich, Germany; ^4^Institute for Stroke and Dementia Research, University Hospital of Munich, Ludwig- Maximilians University Munich, Munich, Germany; ^5^Graduate School of Systemic Neurosciences (GSN), Munich, Germany; ^6^Faculty of Biology, Master of Science Program in Molecular and Cellular Biology, Ludwig-Maximilians-Universität München, Planegg, Germany; ^7^Department of Neuropathology, Regensburg University Hospital, Regensburg, Germany; ^8^Department of Cardiology, University Hospital of Munich, LMU Munich, Munich, Germany; ^9^SyNergy, University of Munich, Munich, Germany; ^10^German Cancer Consortium (DKTK), Partner Site Munich, German Cancer Research Center (DKFZ), Heidelberg, Germany; ^11^DZNE – German Center for Neurodegenerative Diseases, Munich, Germany

**Keywords:** TSPO, PET, glioblastoma, mouse model, SB28

## Abstract

**Introduction:**

The 18 kDa translocator protein (TSPO) receives growing interest as a biomarker in glioblastoma. Mouse models can serve as an important tool for the investigation of biomarkers in glioblastoma, but several glioblastoma models indicated only low TSPO-PET signals in contrast to high TSPO-PET signals of human glioblastoma. Thus, we aimed to investigate TSPO-PET imaging in the syngeneic immunocompetent SB28 mouse model, which is thought to closely represent the tumor microenvironment (TME) of human glioblastoma.

**Methods:**

Dynamic TSPO-PET/CT imaging was performed for 60 min after injection of 13.6 ± 4.2 MBq [^18^F]GE-180. Contrast enhanced CT (ceCT) was acquired prior to PET and served for assessment of tumor volumes and attenuation correction. SB28 and sham mice were imaged at an early (week-1; *n* = 6 SB28, *n* = 6 sham) and a late time-point (week-3; *n* = 8 SB28, *n* = 9 sham) after inoculation. Standard of truth *ex vivo* tumor volumes were obtained for SB28 mice at the late time-point. Tracer kinetics were analyzed for the lesion site and the carotid arteries to establish an image derived input function (IDIF). TSPO-PET and ceCT lesion volumes were compared with *ex vivo* volumes by calculation of root-mean-square-errors (RMSE). Volumes of distribution (VTmax/mean) in the lesion were calculated using carotid IDIF and standardized uptake values (SUVmax/mean) were obtained for a 40–60 min time frame.

**Results:**

Higher uptake rate constants (K1) were observed for week-1 SB28 tumor lesions when compared to week-3 SB28 tumor lesions. Highest agreement between TSPO-PET lesion volumes and *ex vivo* tumor volumes was achieved with a 50% maximum threshold (RMSE-VT: 39.7%; RMSE-SUV: 34.4%), similar to the agreement of ceCT tumor volumes (RMSE: 30.1%). Lesions of SB28 mice had higher PET signal when compared to sham mice at week-1 (VTmax 6.6 ± 2.9 vs. 3.9 ± 0.8, *p* = 0.035; SUVmax 2.3 ± 0.5 vs. 1.2 ± 0.1, *p* < 0.001) and PET signals remained at a similar level at week-3 (VTmax 5.0 ± 1.6 vs. 2.7 ± 0.8, *p* = 0.029; SUVmax 1.9 ± 0.5 vs. 1.2 ± 0.2, *p* = 0.0012). VTmax correlated with SUVmax (*R*^2^ = 0.532, *p* < 0.001).

**Conclusion:**

TSPO-PET imaging of immunocompetent SB28 mice facilitates early detection of tumor signals over sham lesions. SB28 tumors mirror high TSPO-PET signals of human glioblastoma and could serve as a valuable translational model to study TSPO as an imaging biomarker.

## Introduction

In years, the 18 kDa translocator protein (TSPO) emerged as an important imaging target in glioma and the *in vivo* detection of TSPO is feasible with positron emission tomography (PET) and single photon emission computed tomography (SPECT) ligands. TSPO is not only expressed in myeloid cells of the tumor microenvironment (TME) but also in tumor cells in dependence of the glioma grade (1). Despite the expression by various cell types, TSPO could serve as a potential imaging biomarker to characterize the immunosuppressive phenotype of glioblastoma during immunomodulatory treatment (2). Furthermore, due to the assumed strong elevation of TSPO in the infiltration zone (1), this biomarker may have potential to identify brain regions that are subject to tumor recurrence. It was already shown that spatial volumes of TSPO-PET add complementary information to amino acid PET since the spatial agreement of both imaging targets was rather poor (3). Ultimately, TSPO agonists or antagonists could also act as direct modulators of TSPO expression in gliomas (4) and such therapies would profit from monitoring of altered target expression by TSPO-PET. The value of TSPO imaging in glioma was already determined in prognostication and detection of tumor heterogeneity (5, 6). For back translation and mechanistic elucidation of observations in humans as well as for TSPO biomarker monitoring during experimental testing of therapeutics, animal models provide a tremendous value across brain diseases (2, 7, 8). First experimental glioblastoma investigations with murine GL261 in immunocompetent mice (9), human P3 in immunodeficient mice (10), and human U87 in immunodeficient rats (11) indicated feasibility of TSPO PET monitoring by different radiotracers. However, several caveats were observed in these studies. First, the PET signal related to inflammatory response caused by the inoculation was considerable when compared to the magnitude of the TSPO-PET signal in GL261 tumors, especially at early time-points (9). Furthermore, glioblastoma mouse models using primary human tumor cells have limited translational impact since the TME is impacted by immunodeficiency of the host.

Hence we endeavored to investigate TSPO-PET imaging in the syngeneic SB28 glioblastoma mouse model since this cell line can be implanted in immunocompetent mice (12) and is thought to closely resemble the human TME (13). We compared TSPO-PET in mice with SB28 tumors against sham injection at early and late time-points after inoculation. Thresholds for delineation of the TSPO-PET derived tumor volume were evaluated in comparison to contrast enhanced CT (ceCT) relative to *ex vivo* standard of truth. Furthermore, we performed a detailed analysis of different TSPO-PET quantification approaches including image derived input function (IDIF) and standardized uptake values (SUV) of a late imaging window.

## Materials and methods

### Study design

The aim of the study was to investigate TSPO-PET in the syngeneic immunocompetent SB28 glioblastoma mouse model. For this purpose, we obtained 60 min dynamic TSPO-PET after [^18^F]GE-180 injection in mice with tumor cell or sham injection at an early (week-1) and a late (week-3) time-point post inoculation in a cross-sectional design. The majority of mice was sacrificed after the TSPO-PET scan to perform single cell Radiotracing (data not presented in this manuscript), while two mice imaged at week-1 received an additional week-3 TSPO-PET imaging session. PET quantification in the lesion was performed by calculation of volumes of distribution (VT) with the carotid time-activtiy-curve used for an IDIF. Furthermore, SUV were obtained from the late 40–60 min time frame. ceCT lesion volumes were segmented with a unified threshold and optimal TSPO-PET thresholds were determined according to congruence with a standard of truth *ex vivo* volume at week-3. We performed a group wise comparison of TSPO-PET VT and SUV (max and mean) between SB28 and sham mice at both time-points. Furthermore, VTmax/mean were correlated with simplified quantification (SUVmax/mean).

### Animals

All animal experiments were performed in compliance with the National Guidelines for Animal Protection, Germany and with the approval of the regional animal committee (Regierung von Oberbayern) and overseen by a veterinarian. All animals were housed in a temperature- and humidity-controlled environment with a 12-h light–dark cycle, with free access to food (Ssniff, Soest, Germany) and water.

Eight-week-old C57BL/6 mice were obtained from Charles River (Sulzfeld, Germany) and acclimated for 1 week. At day 0, approximately half of the mice were inoculated with 100,000 SB28-GFP cells suspended in DMEM (Merck, Darmstadt, Germany) (GBM mice, *n* = 14) or saline (sham mice, *n* = 15). For inoculation, mice were anesthetized with i. p. injections of 100 mg/kg ketamine 10% and 10 mg/kg xylazine 2% in 0.9% NaCl. Anesthetized mice were immobilized and mounted onto a stereotactic head holder (David Kopf Instruments, Tujunga, CA, USA) in the flat-skull position. After surface disinfection, the skin of the skull was dissected with a scalpel blade. The skull was carefully drilled with a micromotor high-speed drill (Stoelting Co., Wood Dale, IL, USA) 2 mm posterior and 1 mm left of the bregma. By stereotactic injection, 1 × 10^5^ cells or 2 μL saline applied with a 10 μL Hamilton syringe (Hamilton, Bonaduz, Switzerland) at a depth of 2 mm below the drill hole. Cells were slowly injected within 1 min and after a settling period of another 2 min the needle was removed in 1 mm steps per minute. After that, the wound was closed by suturing. Mice were checked daily for tumor-related symptoms and sacrificed when tumor burden (i.e., appearance, coordinative deficits, motor symptoms) reached stop criteria (not reached at week-3 in any animal).

### Cell culture

SB28 is a newly developed mouse cell line, which does not express detectable CD40, and represents a weakly immunogenic glioma model (12). SB28-GFP were cultured in DMEM containing MEM non-essential amino acids (1x), 1% Penicillin-Streptomycin solution (Thermo Fisher Scientific, Waltham, MA, USA) and 10% fetal bovine serum (FBS, Biochrome, Berlin, Germany). Cell cultures were maintained in the incubator at 37°C in humidified and 5% CO_2_-conditioned atmosphere. Cells were passaged when the cell density in the flask reached 80% confluence.

### Radiosynthesis

Automated production of [^18^F]GE-180 was performed on a FASTlab™ synthesizer with single-use disposable cassettes. The pre-filled precursor vial was assembled on the cassette and the cassette was mounted on the synthesizer according to the set-up instructions. The FASTlab™ control software prompts were followed to run the cassette test and to start the synthesis. No carrier added ^18^F-fluoride was produced via ^18^O(p, n)^18^F reaction by proton irradiation of ^18^O-enriched water and delivered to the ^18^F incoming reservoir. The fully automated manufacturing process consists of the following steps: trapping of ^18^F-fluoride on a QMA cartridge, elution using Kryptofix^®^ 222, potassium hydrogen carbonate, water and acetonitrile, azeotropic drying of ^18^F-fluoride at 120°C for 9 min, labeling of the precursor in MeCN at 100°C for 6 min, dilution of the crude product with water, tC18 cartridge based purification by use of 20 mL 40% (v/v) Ethanol and 11.5 mL 35% (v/v) Ethanol, elution of the product with 3.5 mL 55% (v/v) Ethanol and final formulation with phosphate buffer. RCY 39 ± 7% (*n* = 16) non d. c., synthesis time 43 min, RCP ≥ 98%.

### PET imaging and reconstruction

All small animal positron emission tomography (μPET) procedures followed an established standardized protocol for acquisition and post-processing (14, 15). Starting with the injection of [^18^F]GE-180 (13.6 ± 4.2 MBq), PET data were acquired for 60 min to measure cerebral TSPO expression using a small animal Mediso PET/CT system (Mediso Ltd., Muenster, Germany). All small-animal PET experiments were performed with isoflurane anesthesia (1.5% at time of tracer injection and during imaging; delivery 3.5 L/min). The PET reconstruction procedure was an Ordered Subsets Expectation Maximization (OSEM-3D) algorithm with decay correction, scatter correction, attenuation correction, dead time correction, and sensitivity normalization. An x-ray computed tomography scan enabled the attenuation correction. Contrast medium (0.3 mL, Iomeprol 300 M) was injected 5 min before the CT scan. The resulting PET images had 212 × 212 × 235 voxels of 0.4 × 0.4 × 0.4 mm^3^. Data were binned to a total of 28 frames, consisting of 6 × 10 s, 6 × 30 s, 6 × 60 s, and 10 × 300 s. The resulting CT images had 972 × 972 × 930 voxels of 0.12 × 0.12 × 0.12 mm^3^.

### Positron emission tomography preprocessing and quantification

All PET analyses were performed using PMOD (V3.4 PMOD Technologies, Basel, Switzerland). We calculated volume of distribution (VT) images with an IDIF (16) using the methodology described by Logan et al. implemented in PMOD (17). The blood input curve was obtained from a standardized bilateral VOI placed in both carotid arteries (18). A maximum error of 10% and a VT threshold of 0 were selected for modeling of the full dynamic imaging data with a one-tissue compartment model. Time of the linear fit was set flexible (t*) and uptake rate constant K_1_ and dissociation rate constant K_2_ were obtained for the lesion. VT images of the whole mouse were generated. Non-linear PET coregistration (VT and 40–60 min SUV) of the brain was performed via the CT to a brain CT template, with spatial normalization parameters equal to previously described PET template coregistration (15).

The PET lesion volume of interest in SB28 mice was obtained in each animal as described below. For kinetic modeling analysis, a manually drawn individual lesion sphere of 3 mm^3^ served for assessment of PET tracer uptake in SB28 tumors. For sham mice, a standardized 2 mm^3^ sphere at lesion site. The smaller sham-VOI was used in order to avoid spill over from adjacent brain structures like skull whereas the larger sphere in SB28 tumors enhanced the probability to include the majority of the tumor mass in mice with largest glioblastoma.

### TSPO-PET, ceCT and standard-of-truth volume assessments

Tumors of all eight SB28 mice at week-3 were manually dissected from the brain parenchyma after the PET scan and the individual standard-of-truth volume was determined by a microbalance and given specific gravity of tumor tissue. Here, an assumed specific gravity of 1.0 g/cm^3^ allowed assessment of tumor volumes by the wet weight of the tumor (19). ceCT volumes of all SB28 tumors were obtained using a threshold of 30 Hounsfield Units (HU), after cropping extracerebral structures. TSPO-PET lesion volumes were determined using%-thresholds (40–80%) of the maximum tumor uptake within the 3 mm^3^ sphere (separately for VT and SUV images). The optimal PET VOI was used for further VTmax/mean and SUVmax/mean analysis. For additional histological characterization (core vs. infiltration zone) of early- and late-stage SB28 tumor volumes, two independent (day-6, day-26) SB28 tumors were assessed with Hematoxylin-eosin (HE) staining. To this end, the mouse brains were stored in 4% PFA at 4°C for 12 h for fixation. Subsequently, brains were embedded in Cryomatrix and frozen with liquid nitrogen. The mouse brains were cut into 20 μm sagittal slices using a sliding vibratome, slices were collected into 24-well plates filled with PBS and finally mounted onto glass slides and air dried for 15 min. HE staining was performed according to standard protocol. The Cavalieri method was applied to estimate core and infiltration zone tumor volumes using Fiji (based on Image-J2, NIH, Bethesda, MD, USA) (20).

### Cryosectioning and immunofluorescence staining

One extracted week-3 tumor was incubated in 30% sucrose overnight at room temperature (RT) for dehydration. The tumor was embedded in OCT and frozen at –80°C for 1 h. Cryostat chamber and head temperatures were set to –20°C. Sample was rested in –20°C for half an hour inside the chamber, attached to the freezing head and placed to sample holder. Sample was sectioned in 10 μm sections and placed onto super frost microscope slides to be labeled. Tissue sections on glass slides were blocked with 0.2% TritonX-100, 10% DMSO, and 10% goat serum in 0.1 M PBS for 1 h at RT. They were incubated with primary antibody [Recombinant Anti-PBR antibody (EPR5384) (ab109497), CD11b Antibody, anti-mouse, REAfinity™ (130-113-806)] and GFP labeled cancer cells were conjugated with Chromotek GFP-Booster Atto647N (GBA647N) in 0.2% Tween-20, 5% DMSO, 5% goat serum, 0.001% heparin in 0.1 M PBS overnight at 4°C. They were washed with 0.2% Tween-20, 0.001% heparin in 0.1 M PBS for 3 × 5 min. After washing, they were incubated with secondary antibody [Goat anti-Rabbit IgG (H + L) Highly Cross-Adsorbed Secondary Antibody, Alexa Fluor 647 (A21245), Goat anti-Rabbit IgG (H + L) Highly Cross-Adsorbed Secondary Antibody, Alexa Fluor 568 (A-11036)] in a solution composed of 0.2% Tween-20, 5% goat serum, 0.001% heparin in 0.1 M PBS for 0.5–1 h. They were washed in 0.2% Tween-20, 0.001% heparin in 0.1 M PBS for 3 × 5 min. Next, the slides were incubated in Invitrogen™ ProLong™ Gold Antifade Mountant with DAPI (P36931) and then covered with coverslips. Samples were imaged after overnight incubation at 4°C.

### Confocal microscopy

Confocal microscopy was performed on Leica SP8 using 10x, 40x, and 63x objectives. Images were obtained as 8-bits with HC PL APO CS2 40 × /1.30 NA objective, 1,024 × 1,024 resolution, 200 Hz, at 20–25°C. Tile scans were obtained with z-step size of 1 μm to allow 3D reconstruction of tissue for depth of 10 μm.

### Statistics

All statistical analyses were conducted with GraphPad Prism (V9.4.0, GraphPad Software LCC). A *p*-value < 0.05 was considered as significant. Area under the curve (AUC) values of time-activtiy-curves as well as K_1_ and K_2_ estimates were compared between SB28 and sham mice at week-1 and week-3 using one-way ANOVA including a Tukey *post-hoc* test. RMSE were compared between PET threshold and ceCT derived tumor volumes by considering individual absolute deviations from the standard of truth by a paired *t*-test. Tumor volumes between time-points and groups were compared by an unpaired *t*-test. VTmax/mean and SUVmax/mean values were compared between groups of SB28 and sham mice at week-1 and week-3 using one-way ANOVA including a Tukey *post-hoc* test. VTmax/mean and SUVmax/mean values were correlated using Pearson’s coefficient of correlation (R).

## Results

### [^18^F]GE-180 tracer kinetics in SB28 and sham lesions

**FIGURE 1:**
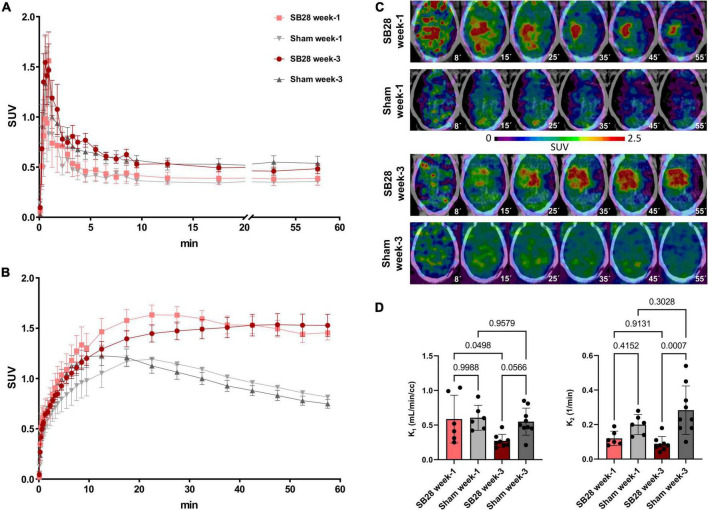
[^18^F]GE-180 tracer kinetics in SB28 and sham mice. Time-activity-curves (normalized to standardized uptake values, SUV) of **(A)** the carotid arteries and **(B)** the lesion site in SB28 and sham mice at early (week-1) and late (week-3) time-points. Time-activity-curves show the average curve for each group of animals (SB28 and sham) at both time-points. **(C)** Axial slices of [^18^F]GE-180 TSPO-PET at different time frames of the 60 min scan duration are shown upon a CT template for representative mice. The median times (minutes) of the illustrated frames are provided below the images. Please note the faster tracer uptake and the higher washout in the early SB28 mouse when compared to the late SB28 mouse. **(D)** Comparison of the uptake rate constant K_1_ and the dissociation rate constant K_2_ between SB28 and sham mice at early (week-1) and late (week-3) time-points. K_1_ and K_2_ were derived from a one tissue compartment model using the image derived carotid artery input function. *P*-values are indicated for comparisons of SB28 and sham mice per time-point and for the comparison of week-1 (SB28 *n* = 6, sham *n* = 6) and week-3 (SB28 *n* = 8, sham = 9) within groups.

Dynamic PET imaging allowed to delineate the [^18^F]GE-180 signal in whole blood using bilateral carotid regions of interest (Figure 1A). The obtained carotid IDIF was similar between SB28 and sham mice at week-1 (AUC: + 9%; *p* = 0.654) and week-3 (AUC: –5%; *p* = 0.663). Carotid IDIF was lower at week-1 when compared to week-3, regardless of SB28 or sham inoculation (AUC: –27%, *p* = 0.0056). The [^18^F]GE-180 signal in SB28 tumors showed peak uptake at 25 min p.i. at week-1 and a late plateau (∼40–60 min p.i.) at week-3 (Figure 1B). SB28 mice at week-1 indicated a higher washout from the lesion site when compared so SB28 mice at week-3 (Figure 1C). K_1_ of SB28 tumors was higher at week-1 when compared to week-3 (0.59 mL/min/cc vs. 0.27 mL/min/cc; *p* = 0.0498; Figure 1D) whereas K_2_ was not significantly different between both time-points (0.12 1/min vs. 0.09 1/min; *p* = 0.913; Figure 1D). Sham lesions indicated higher washout when compared to tumor lesions regardless of the investigated time-point, reaching significance at week-3 (K2 week-1: 0.20 1/min vs. 0.12 1/min, *p* = 0.415/K2 week-3: 0.028 1/min vs. 0.09 1/min, *p* = 0.0007; Figure 1D).

### Comparison of TSPO-PET derived and ceCT derived SB28 tumor volumes

**FIGURE 2:**
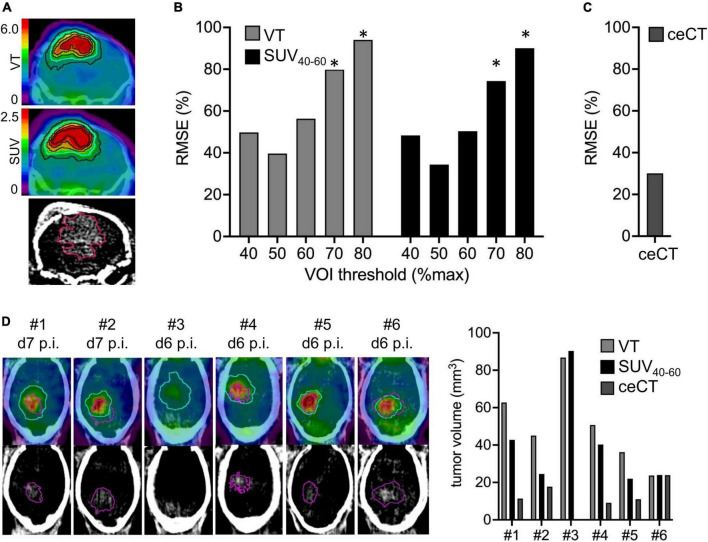
Assessment of lesion volumes by TSPO-PET and contrast enhanced CT (ceCT). **(A)** TSPO-PET and ceCT images illustrate lesion volume definition for a SB28 tumor at week-3. Isocontours represent different %max thresholds (blue = 50%) of TSPO-PET and the 30HU ceCT threshold. PET derived lesion volumes with different thresholds **(B)** and ceCT lesion volumes **(C)** were compared to standard-of-truth *ex vivo* tumor volume assessment in week-3 SB28 mice by calculation of root-mean-square-errors (RMSE%). **(D)** Direct comparison of lesion volume assessment between PET and ceCT in week-1 SB28 mice (*n* = 6). Axial slices show TSPO-PET VT and ceCT. The determined optimal 50% PET threshold (blue) was used for assessment of TSPO-PET lesion volumes. The ceCT threshold is shown in purple. Mouse #3 did not indicate a ceCT-positive lesion volume. VOI, volume of interest; p.i., post inoculation; VT, volume of distribution. *Indicates significantly higher RMSE or volume of PET vs. ceCT (*p* < 0.05).

We analyzed the comparability of TSPO-PET and ceCT for assessment of SB28 lesion volumes *in vivo*. Lesion volumes obtained from thresholded TSPO-PET signals and ceCT were compared against standard of truth *ex vivo* tumor volumes at week-3 (Figure 2A). The standard of truth volume at week-3 was 85 ± 55 mm^3^ (range: 26–140 mm^3^; Table 1). Quantitative histological assessment of week-1 tumor composition indicated a small compact HE-positive tumor core but a large infiltration zone, whereas a late-stage week-3.5 tumor was characterized by a large HE-positive lesion with high density and only minor infiltration zone (Supplementary Figure 1).

**TABLE 1:** Overview on study cohorts and main results in SB28 and sham mice.

Model	N	Days post inoculation	Body weight (g)	Dose (MBq)	VTmax (0–60)	SUVmax (40–60)	VTmean (0–60)	SUVmean (40–60)	*Ex vivo* tumor volume (wet weight transformed to mm^3^)	TSPO-PET VT lesion volume (mm^3^)	TSPO-PET SUV lesion volume (mm^3^)	ceCT lesion volume (mm^3^)
SB28 week-1	6	6.3 ± 0.5	20.6 ± 0.8	11.6 ± 2.3	6.6 ± 2.9*	2.3 ± 0.5***	4.1 ± 1.7	1.4 ± 0.3**	n.a.	51 ± 22	41 ± 26	12 ± 8
SB28 week-3	8	18.5 ± 0.5	19.6 ± 3.4	15.0 ± 7.1	5.0 ± 1.6*	1.9 ± 0.5**	3.1 ± 0.9	1.2 ± 0.3**	85 ± 55	80 ± 38	86 ± 37	75 ± 59
Sham week-1	6	8.3 ± 2.6	21.3 ± 0.4	13.5 ± 2.7	3.9 ± 0.8	1.2 ± 0.1	2.9 ± 0.5	0.9 ± 0.1	n.a.	n.a.	n.a.	n.a.
Sham week-3	9	21.9 ± 2.4	22.1 ± 1.1	13.9 ± 6.7	2.7 ± 0.8	1.2 ± 0.2	2.0 ± 0.6	0.8 ± 0.1	n.a.	n.a.	n.a.	n.a.

VT, volume of distribution; SUV, standardized uptake value; TSPO-PET, 18 kDa translocator protein positron-emission-tomography; ceCT, contrast enhanced computed tomography. Significant differences between SB28 and sham mice per time-point are indicated by * < 0.5, ** < 0.01, *** < 0.001.

RMSE of TSPO-PET VT derived lesion volumes at the late week-3 imaging time-point were lowest for a 50%-max threshold (Figure 2B). Individual deviations of TSPO-PET derived lesion volumes were similar to deviations of ceCT derived lesion volumes (Figure 2C) for the VT threshold of 50% but higher RMSE were observed for PET thresholds ≥ 70% (all *p* < 0.05). Late static TSPO-PET SUV derived lesion volumes showed similar performance with best standard-of-truth tumor volume representation at a 50%-max threshold and higher RMSE of PET when compared to ceCT for PET thresholds ≥ 70% (all *p* < 0.05).

These optimized thresholds were used for comparison of TSPO-PET and ceCT derived SB28 lesion volumes at the early imaging time-point. All SB28 mice at week-1 indicated larger TSPO-PET lesion volumes when compared to CT lesion volumes (Figure 2D). Noteworthy, even the individual mouse that did not show any relevant contrast enhancement at week-1 after inoculation showed an elevated TSPO-PET signal and a large PET derived lesion volume (VT volume 90 mm^3^/SUV volume: 87 mm^3^) at the lesion site. TSPO-PET (VT derived: 51 ± 22 mm^3^ vs. 80 ± 38 mm^3^, *p* = 0.121; SUV derived: 41 ± 26 mm^3^ vs. 86 ± 37 mm^3^, *p* = 0.026) and ceCT (12 ± 8 mm^3^ vs. 75 ± 59 mm^3^, *p* = 0.026) derived lesion volumes were larger at week-3 when compared to week-1 (Table 1) and week-1 TSPO-PET derived lesion volumes exceeded week-1 ceCT derived lesion volumes (p_*SUV*_ = 0.028; p_*VT*_ = 0.002).

### TSPO-PET assessment in SB28 mice in comparison to sham mice

**FIGURE 3:**
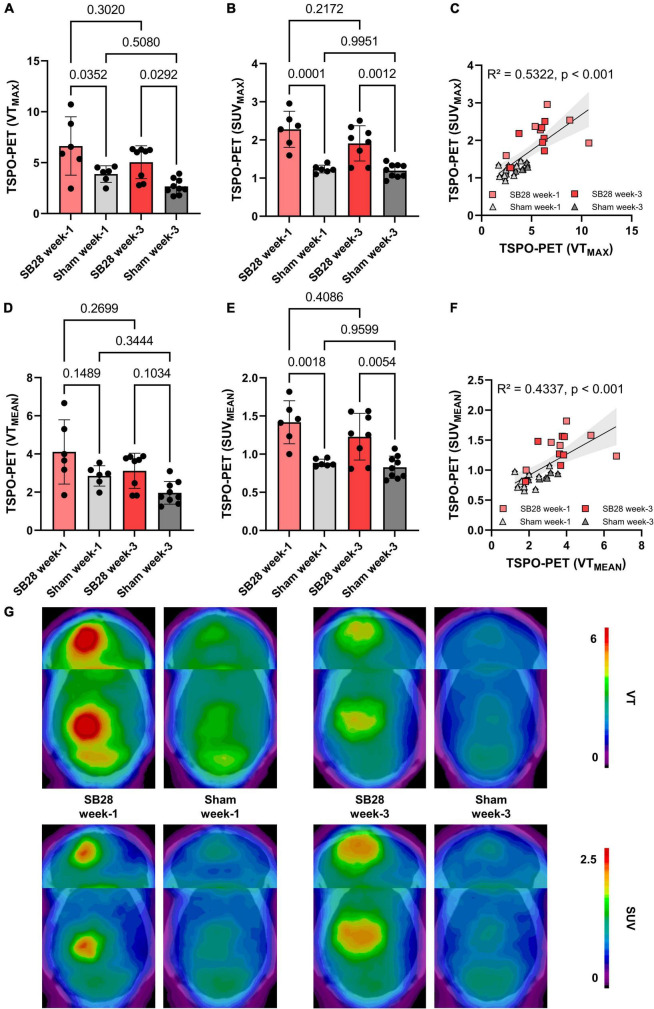
Quantitative assessment of TSPO in SB28 and sham mice. **(A–C)** Maximum volumes of distribution (VT) and standardized uptake values (SUV) of the lesion site in SB28 and sham mice at week-1 and week-3 as well as their correlation. Maximum values were obtained using the hottest voxel in individual 50% threshold tumor VOIs or a standardized sham VOI. **(D–F)** Mean volumes of distribution (VT) and standardized uptake values (SUV) of the lesion site in SB28 and sham mice at week-1 and week-3 as well as their correlation. Mean values were obtained using the average signal intensity of the individual 50% threshold tumor VOIs or a standardized sham VOI. *P*-values are indicated for comparisons of SB28 and sham mice per time-point and for the comparison of week-1 and week-3 within groups. **(G)** Axial planes show group average images of SB28 and sham mice at both time-points upon a CT template. To avoid bias in visualization, *n* = 4 week-1 and *n* = 6 week-3 SB28 mice were used for average images due to exclusion of two mice per time-point with a deviating center of tumor localization.

TSPO-PET quantification (max and mean values) obtained from optimal tumor regions of interest in SB28 mice and standardized regions of interest in sham mice was compared between the study groups (Table 1). SB28 mice had higher VTmax (6.6 ± 2.9 vs. 3.9 ± 0.8, *p* = 0.035, Figure 3A) and SUVmax (2.3 ± 0.5 vs. 1.2 ± 0.1, *p* < 0.001, Figure 3B) at week-1 when compared to sham. Higher VTmax (5.0 ± 1.6 vs. 2.7 ± 0.8, *p* = 0.029, Figure 3A) and SUVmax (1.9 ± 0.5 vs. 1.2 ± 0.2, *p* = 0.0012, Figure 3B) were also observed in SB28 tumors when compared to sham lesions at week-3. Mean VT differences between SB28 and sham mice did not reach significance for both time-points (Figure 3D), whereas mean SUV of SB28 mice were significantly higher than SUV of sham mice at week-1 and week-3 (Figure 3E). There were no significant differences of SB28 lesion VT and SUV between week-1 and week-3. This was confirmed by additional serial TSPO-PET imaging of two mice which indicated similar SUVmax and SUVmean values at week-1 and week-3 (Supplementary Figure 2). Strong TSPO expression of tumor cells and tumor-associated immune cells was confirmed by qualitative immunofluorescence imaging of a week-3 tumor (Supplementary Figure 3). CoV of VT were higher when compared to SUV (30 ± 9% vs. 17 ± 7%, *p* = 0.004). VT and SUV were significantly correlated (VTmax/SUVmax: *R*^2^ = 0.532, *p* < 0.001; Figure 3C; VTmean/SUVmean: *R*^2^ = 0.434, *p* < 0.001; Figure 3F). The cerebellum showed known high TSPO-PET signals without significant VTmean and SUVmean differences between SB28 and sham mice at both time-points (Supplementary Figure 4). Cerebellar TSPO-PET VTmean in sham mice showed a decrease from week-1 to week-3 which could be related to a general transient neuroinflammation early after inoculation.

## Discussion

For the first time, we investigated [^18^F]GE-180 TSPO-PET in the SB28 orthotopic glioblastoma model. Using absolute quantification via IDIF, we found strong tracer binding at the tumor site at early (week-1) and late (week-3) time-points after inoculation. Importantly, even at the early time-point, SB28 mice indicated higher TSPO-PET VT and SUV when compared to sham mice, suggesting a promising combination of tracer and mouse model for preclinical TSPO-PET imaging of glioblastoma. Standard-of-truth tumor volumes were resembled best by a TSPO-PET threshold of 50% lesion maximum and similar to contrast enhanced CT.

**FIGURE 4:**
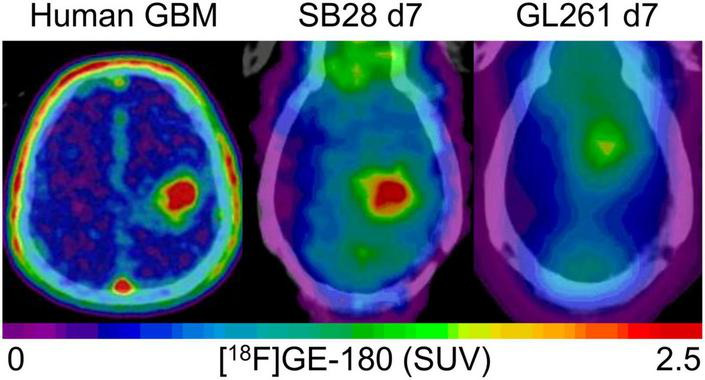
Visual comparison of TSPO enrichment in human glioblastoma and week-1 SB28 and GL261 tumors in immunocompetent mice. The patient example shows a 77 year old male with initial diagnosis of glioblastoma [IDH-wild-type, TERT methylation (+), MGMT methylation (+)], who received a TSPO-PET prior to radiochemotherapy. SB28 tumors at week-1 in immunocompetent mice show similar TSPO-PET signal intensities with human glioblastoma whereas the commonly used GL261 tumor in the same host type indicates only faint TSPO tracer uptake at week-1. Effect sizes (Cohen’s d) of TSPO-PET imaging (SUVmean) at week-1 for the comparison of tumor and sham mice were 2.63 for SB28 mice and 0.01 for GL261 mice (9).

The main goal of the study was to establish and investigate TSPO-PET in the SB28 glioblastoma model. Previous TSPO-PET imaging studies predominantly investigated the GL261 model using [^18^F]DPA-714 (2), [^18^F]PBR111 (21), and [^18^F]GE-180 (9) TSPO-PET, successfully showing the possibility to monitor TSPO expression and tumor growth *in vivo.* The main finding of our investigation was high TSPO-PET values of SB28 lesions, which were distinctly elevated over sham lesions already at week-1 and remained at a high level until week-3. While direct comparisons of PET quantification remain difficult due to lacking standardization, our in house [^18^F]GE-180 study in GL261 mice showed tumor SUVmean increases of only 1.1-fold over sham at week-1 and 1.4-fold at week-2 (9), indicating a distinctly lower TSPO-PET signal when compared to the early week-1 time-point of our current SB28 data (tumor vs. sham: 1.6-fold). This observation may be related to improved resembling of the human TME by SB28 tumors when compared to GL261 tumors (12). Human therapy naïve glioblastoma are characterized by a strong TSPO expression in tumor cells and cells of the TME (1, 22, 23) which translates to a strong TSPO-PET signal when using different TSPO radioligands (23–28). Thus, our data suggest that the immunocompetent SB28 mouse model resembles similar imaging characteristics when compared to TSPO-PET imaging in human glioblastoma (Figure 4). In this regard, monitoring of a invasively growing primary glioma model (P3) in immunodeficient mice by [^18^F]DPA-714 also revealed only low SUV increases over the contralateral hemisphere between week-1 and week-5 (1.1-1.2-fold) (10). Thus, tumor compactness and immune competence of the host animal may have an additional impact on the magnitude of TSPO-PET signals in glioblastoma mouse models. Furthermore, the purpose of the study needs to guide the selection of the appropriate mouse model, which may be defined by high TSPO expression at early stages or several other features. In conclusion, the observed strong early elevations of the TSPO-PET signal in SB28 mice comprise an important feature of the model and potentially provide the opportunity for sensitive TSPO-PET monitoring of early interventions. To underpin this claim, we tested the sensitivity of TSPO-PET in early-stage SB28 tumors against ceCT and confirmed the excellent sensitivity of TSPO-PET by the observation of larger TSPO-PET derived lesion volumes when compared to ceCT derived lesion volumes. As a potential confounder in this comparison, the occurrence of stronger partial volume effects (29) need to be considered for small week-1 lesions, given the low resolution of small animal PET systems. However, it needs to be acknowledged that TSPO is not only expressed by the tumor cells of the dense tumor core but also by tumor cells and TME cells of the large infiltration zone at week-1. Thus, TSPO-PET probably captures larger proportions of SB28 lesions when compared to lesion sites with contrast enhancement. This hypothesis is strengthened by distinctly lower ceCT derived SB28 tumor volumes mice at week-1 when compared to week-3 SB28 tumors, most likely related to increasing blood-brain-barrier disruption during tumor progression. We note that wet weight measurement did not present a suitable surrogate approach of tumor volume assessment for week-1 SB28 tumors, since a precise dissection can be hampered by the detectability of the small lesion size. Thus, our data do not include a direct comparison of TSPO-PET and ceCT derived lesion volumes against week-1 standard of truth tumor volumes. Nonetheless, we found that TSPO-PET derived lesion volumes recapitulated real *ex vivo* tumor volumes at week-3 with equal precision when compared to ceCT. Thus, ceCT is probably not sensitive enough to detect the whole SB28 tumor volume at week-1 but has similar performance when compared to TSPO-PET at week-3. In this regard, TSPO-PET labels viable tumor and TME cells with TSPO expression (1) whereas contrast enhancement occurs in regions with blood-brain-barrier disruption such as tumor necrosis (30). In conclusion, ceCT and TSPO-PET derived lesion volumes should probably be considered as complementary glioma biomarkers (5). As a limitation, we note the lack of head-to-head MRI and immunohistochemistry correlation in the current study since this study is focused on PET quantification whereas cellular signal sources (31) and tumor heterogeneity in conjunction with 3D immunohistochemistry (32) will be addressed in a dedicated investigation. Earlier studies already characterized SB28 mice by histology and reported high density of tumor cells but modest infiltration by immune cells in end stage lesions (13).

We aimed to determine a detailed work-up of the quantification protocol for [^18^F]GE-180 TSPO-PET imaging in the SB28 glioblastoma model. Most earlier PET studies in glioblastoma models applied TBR in late static windows for TSPO-PET imaging (9). However, such approaches do not take alterations in blood flow (33) or pathology related changes in the reference region into account. Thus, we applied a dynamic imaging protocol and used an IDIF for whole blood adjusted quantification of the [^18^F]GE-180 PET signal. IDIF approaches were successfully applied in preclinical models of stroke (34), hippocampal sclerosis (35) and Alzheimer’s disease (18), providing an excellent tool for quantification near to the gold standard of arterial sampling. We validated an IDIF using the carotid arteries since a left ventricle region of interest is affected from spill-over by myocardial tracer uptake (36). Interestingly, we observed slightly lower tracer signal in the carotid arteries at week-1 when compared to week-3, regardless of implanted tumor cells or sham injection. We speculate that a general inflammation and TSPO upregulation in the organism early after surgery may be responsible for reduced tracer availability in blood. Regarding tracer kinetics in the SB28 tumor, we observed a lower uptake rate constant of late stage tumors at week-3 when compared to early stage tumors at week-1. This finding was unexpected since several publications claimed that the [^18^F]GE-180 PET signal is driven by blood-brain-barrier disruption (37, 38). Importantly, the lower uptake rate constant of [^18^F]GE-180 at week-3 was accompanied by an increased contrast enhancement in SB28 tumors when compared to week-1. One explanation could be that an early immune response to tumor infiltration increases the blood-brain-barrier permeability (30). Our findings emphasize the need to consider global and tumor specific effects of tracer kinetics that potentially have an impact on tracer binding to TSPO in brain and tumor. However, we also note that variance (CoV) of VT as assessed with IDIF was higher when compared to SUV. Thus, comparisons of individual SB28 tumor SUV may result in higher statistical power and sensitivity, while VT are convenient to validate results with adjustment for blood flow and tracer plasma availability at the group level.

Among the limitations of the study, we note that detailed time courses of TSPO-PET signals in SB28 and underlying cellular sources of TSPO-PET signals are subject to ongoing investigations. Disentangling the cellular sources of TSPO-PET signals in glioblastoma models will be of tremendous interest since various cell types may contribute to the net TSPO expression and TSPO-PET signal (1).

## Conclusion

TSPO-PET imaging of immunocompetent SB28 mice facilitates early detection of lesion signals and robust increases of TSPO-PET quantification in SB28 over sham mice. TSPO-PET in SB28 mice yields a high potential to study therapeutic effects on TSPO as a glioblastoma biomarker.

## Data availability statement

The raw data supporting the conclusions of this article will be made available by the authors, without undue reservation.

## Ethics statement

This animal study was reviewed and approved by the Regierung von Oberbayern.

## Author contributions

LMB performed the tumor inoculation, performed the PET acquisition, image analysis and interpretation of PET scans, performed the regional PET analyses, and wrote the first draft of the manuscript with input of all co-authors. SVK, JB, and SQ contributed and harvested SB28 cells and interpreted PET data in the context of human glioblastoma imaging. KW, LHK, AH, LG, and AZ participated in preclinical PET acquisition, image analysis, and interpretation. ZIK and SU performed immunohistological analyses and interpretation. LW and MJR interpreted results of SB28 mice in the context of different glioblastoma mouse models and primary human cell lines. PB, DM, JCT, SZ, LB, NLA, and MB contributed to conception and design, interpreting data, and enhancing intellectual content of manuscript. All authors contributed with intellectual content and revised the manuscript.
