# Supplementary material for: 18 kDa translocator protein positron emission tomography facilitates early and robust tumor detection in the immunocompetent SB28 glioblastoma mouse model
Source: Front Med (Lausanne). 2022 Oct 17;9:992993. doi: 10.3389/fmed.2022.992993 (PMC9621314; doi:10.3389/fmed.2022.992993)
Supplement: Supplementary file 1 [file Data_Sheet_1.docx]

**Supplemental Figure 1**

**
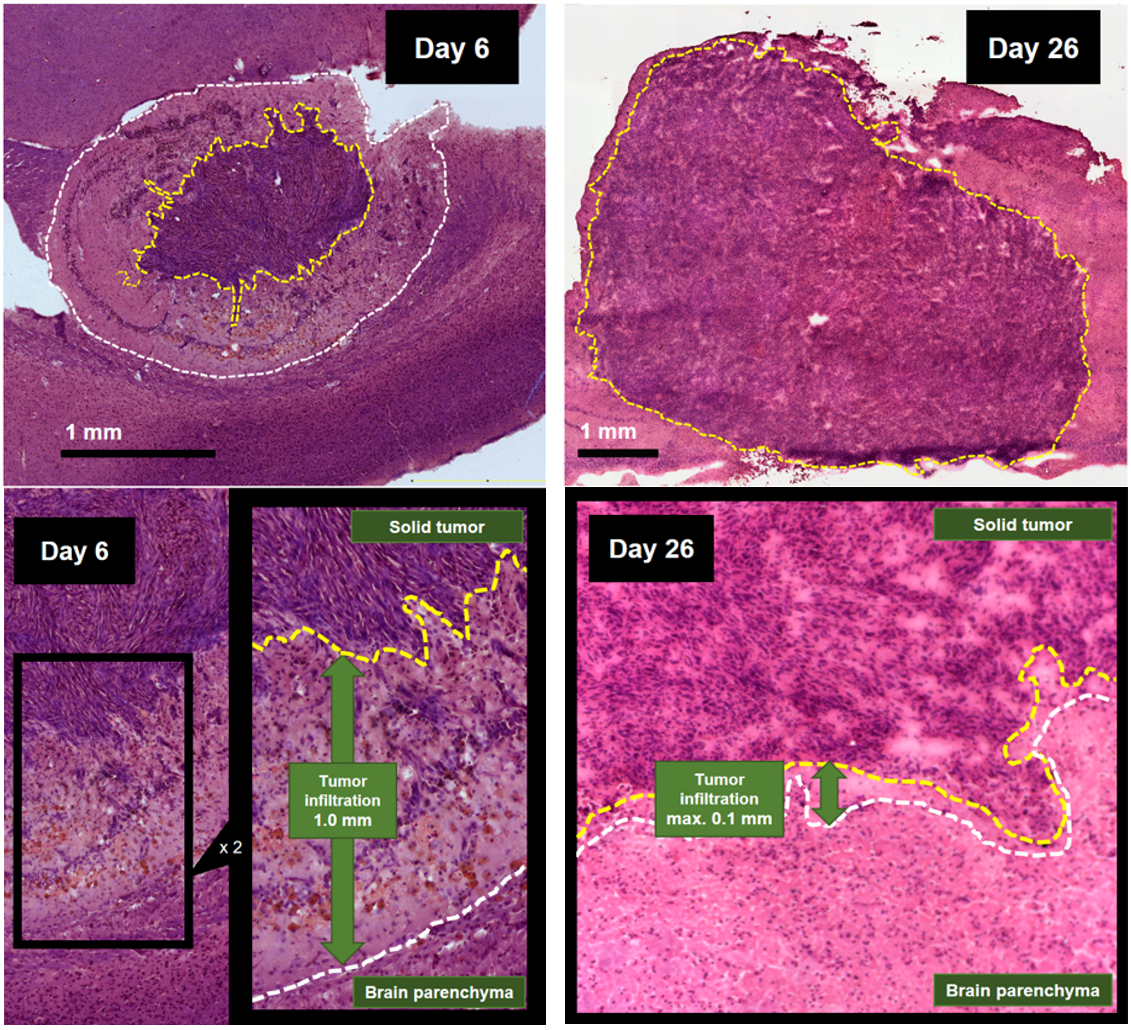
**

**Supplemental Figure 1: Hematoxylin-eosin (HE) volumetric analysis of early- and late-stage SB28 tumors.** The day-6 tumor indicated a small compact HE-positive tumor core but a large infiltration zone, whereas the late-stage day-26 tumor was characterized by a large HE-positive lesion with high density and only minor infiltration zone. Volumetric results: Day 6: Tumor: 7.1 mm^3^ (solid tumor: 3.6 mm^3^ = 50.7%, infiltration zone: 3.5 mm^3^ = 49.3%). Day 26: Tumor 62.8 mm^3^ (solid tumor: 62.8 mm^3^, infiltration zone: not measurable).

**Supplemental Figure 2**

**
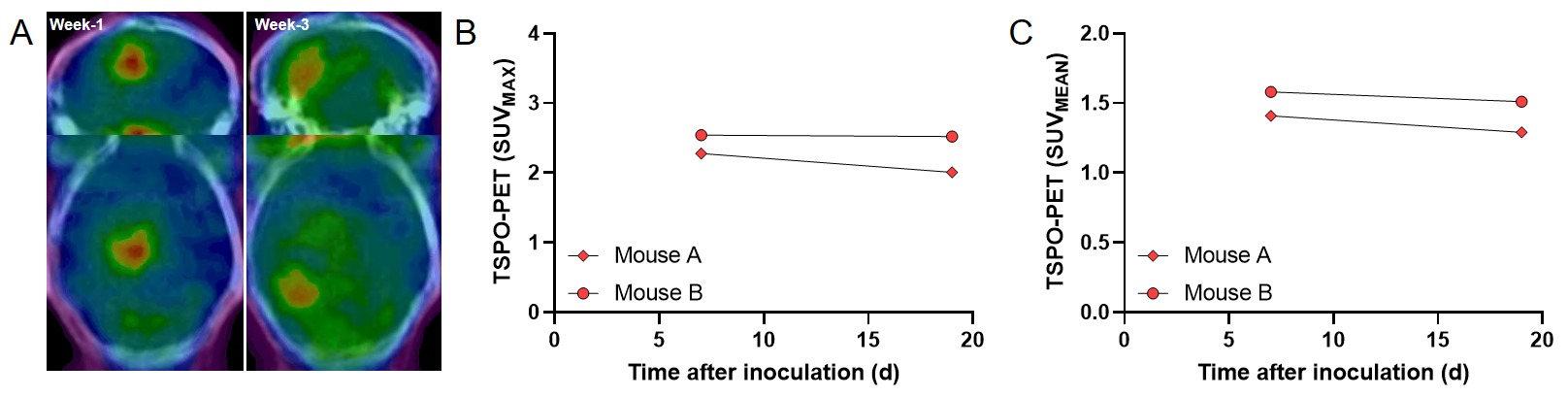
**

**Supplemental Figure 2: Serial TSPO-PET imaging of two SB28 mice.** (**A**) Coronal and axial planes show serial TSPO-PET images of a SB28 mouse at both time-points (week-1 = day 7 / week-3 = day 19) upon a CT template. Note the regional shift of the tumor hotspot during tumor growth. (**B,C**) Quantification of serial TSPO-PET imaging in two SB28 mice.

**Supplemental Figure 3**


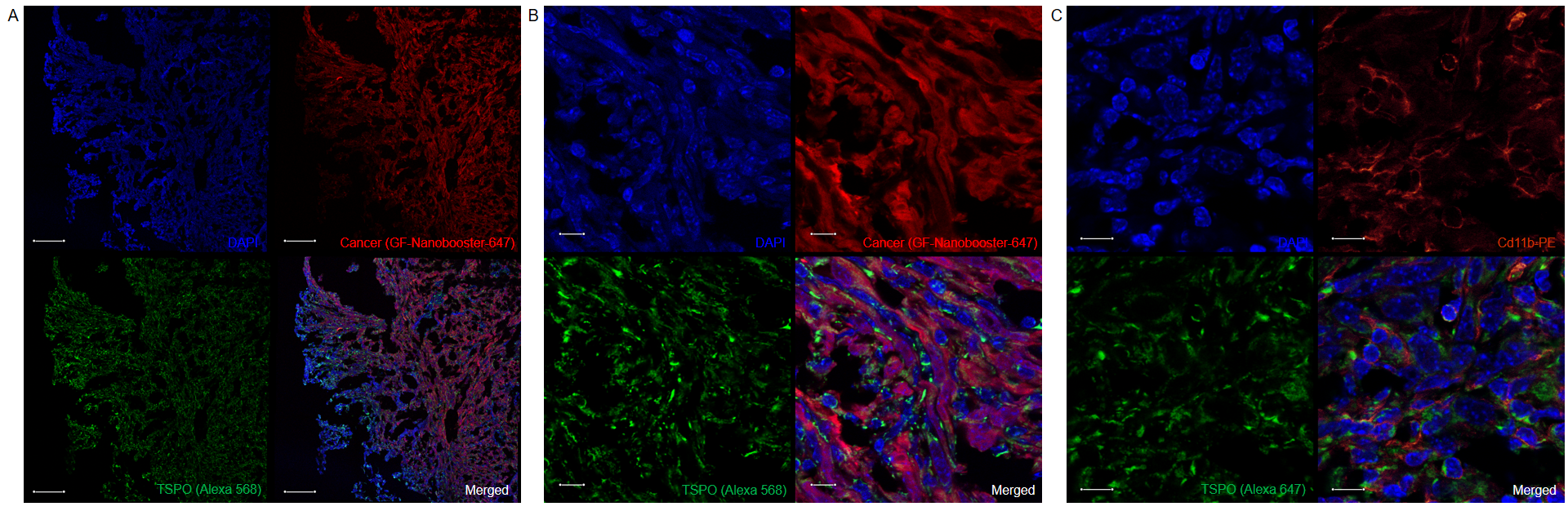


**Supplemental Figure 3: Qualitative immunofluorescence tissue imaging of a week-3 tumor.** (**A**) Strong TSPO labeling of SB28 tumor tissue across all parts of the lesion in an overview section comprising the edge (left: infiltration zone, less GFP-positive cells) and the core of the tumor (right: more GFP-positive cells). DAPI indicates cell cores. Scale bar indicates 100 µm. (**B**) Zoomed section showing co-labeling of GFP-positive tumor cells and TSPO. DAPI indicates cell cores. Scale bar indicates 10 µm. (**C**) Zoomed section showing co-labeling of CD11b-positive immune cells and TSPO. DAPI indicates cell cores. Scale bar indicates 10 µm.

**Supplemental Figure 4**


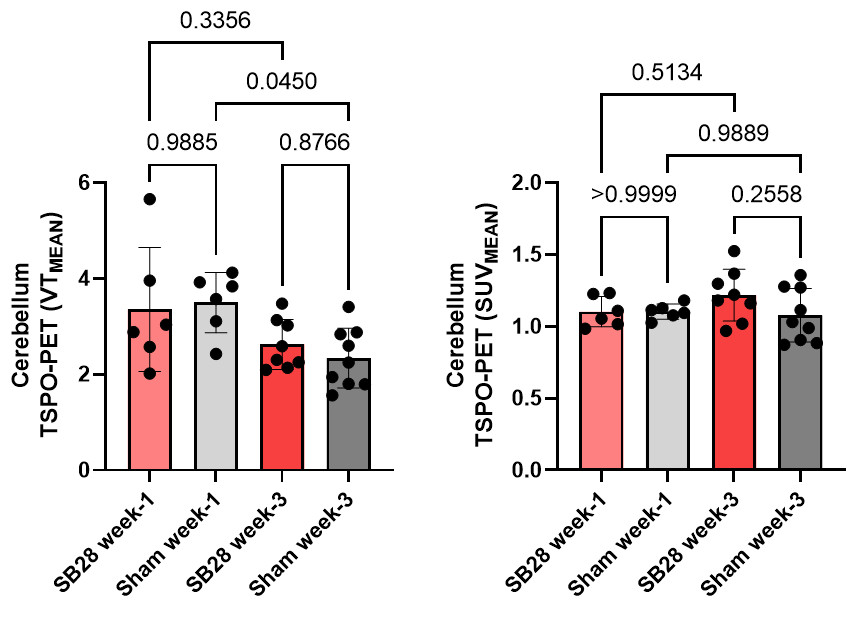


**Supplemental Figure 4: Quantitative assessment of cerebellar TSPO-PET signals in SB28 and sham mice.** Mean volumes of distribution (VT) and standardized uptake values (SUV) of the cerebellum in SB28 and sham mice at week-1 and week-3. Mean values were obtained using a standard VOI of the Mirrione atlas. P values are indicated for comparisons of SB28 and sham mice per time-point and for the comparison of week-1 and week-3 within groups.
